# Evaluation of infection risk and antibiotic exposure in traumatic brain injury patients treated with therapeutic normothermia

**DOI:** 10.1186/cc14526

**Published:** 2015-03-16

**Authors:** J DeGrote, B Heather, J Jancik

**Affiliations:** 1 Hennepin County Medical Center, Minneapolis, MN, USA

## Introduction

The purpose of this study is to assess the rate of confirmed infections, antibiotic exposure, and monitoring practices with normothermia protocol utilization for traumatic brain injury patients. Treatment and prevention of fever is a focus of therapy for patients with severe neurological injury as fever has been identified as an independent risk factor for morbidity and mortality [1].

## Methods

The retrospective chart review analyzed outcomes of maintaining normothermia at 36.5°C versus a similar population without temperature control as a standard of care in patients admitted with traumatic brain injuries defined as a Glasgow Coma Score <8 upon admission. Patients included were 18 to 59 years of age and were mechanically ventilated with intracranial pressure monitoring for greater than 72 hours. The primary outcome evaluated was the number of patients treated for confirmed infections. Secondary outcomes included the antibiotic length of therapy (LOT), antibiotic days of therapy (DOT), number of cultures, and ICU and hospital length of stay (LOS). DOT was defined as the sum of days on which each antibacterial drug was administered.

## Results

A total of 23 patients treated with normothermia and 119 patients in the control group were evaluated between January 2009 and September 2014. The number of patients treated for confirmed infections was similar between groups (normothermia: 73.9%, control: 80%, *P *= 0.803). Empiric antibiotic therapy was more commonly utilized in the normothermia group at 34% versus 20.5% (*P *= 0.173). Antibiotic LOT and DOT were 13.8 versus 10.8 days (*P *= 0.157) and 18.3 versus 16.2 days (*P *= 0.575) in the normothermia versus control groups, respectively. Total culture rate was lower in the normothermia group with 13.2 versus 8.78 (*P *= 0.0002) cultures per patient. No significant difference in hospital LOS (normothermia: 23 days, control: 18 days, *P *= 0.158) or ICU LOS (normothermia: 17 days, control: 15 days, *P *= 0.185) was demonstrated.

## Conclusion

Rates of confirmed infections and number of antibiotic days were similar between the normothermia and control groups, suggesting that normothermia does not increase infection risk. However, the number of cultures obtained in the control group was significantly greater than the normothermia group with a trend toward increased empiric antibiotic use.
